# Cultural competency and inclusion training for healthcare professionals: a systematic review to inform training for health research professionals

**DOI:** 10.1186/s12913-026-14263-1

**Published:** 2026-03-03

**Authors:** Emma Beeston, Anisha Chitrakar, Koby Acheampong, Laura J Gray, Sally Singh, Esther Moss, Natalie Darko

**Affiliations:** 1 Global Health, Lifestyle and Metabolic Health Division, University of Leicester, Leicester, UK; 2 Cancer Sciences Division, University of Leicester, Leicester, UK; 3 Respiratory Sciences Division, University of Leicester, Leicester, UK; 4 Public Health and Epidemiology, University of Leicester, Leicester, UK; 5 NIHR Leicester Biomedical Research Centre, University of Leicester, Leicester, UK; 6 NIHR Applied Research Collaboration East Midlands, University of Leicester, Leicester, UK; 7 Leicester British Heart Foundation Centre of Research Excellence, University of Leicester, Leicester, UK

**Keywords:** Cultural competency, Cultural humility, Diversity and inclusion, Cultural competency training, Healthcare professionals, Health research professionals, Systematic review

## Abstract

**Background:**

Cultural competency and inclusion training are used to address inequalities in care and strengthen equitable health systems. While well established for healthcare professionals (HCPs), the role and design of such training for health research professionals (HRPs) remain underexplored and under-reported in peer-reviewed literature. This review focused on published training interventions for practising HCPs, excluding pre-service training embedded in medical or nursing curricula, in order to inform the development of tailored training for HRPs.

**Methods:**

A systematic review was performed to synthesise the published literature on cultural competency, diversity and inclusion training delivered primarily to healthcare professionals, to identify transferable elements relevant to health research professionals. A protocol was registered with the University of Leicester 10.25392/leicester.data.28284974.v1 Searches of four databases were completed in December 2024. Studies were screened against predefined criteria by two reviewers, with risk of bias assessed using the relevant Joanna Briggs Institute (JBI) checklists. A thematic narrative synthesis was undertaken to appraise and integrate findings across a broad and heterogeneous evidence base.

**Results:**

Twenty-one studies met inclusion criteria. Training formats included face-to-face (38.1%), online (14.3%) and hybrid (47.6%). Most reported short-term improvements in knowledge or attitudes, but few demonstrated sustained behaviour change and only one study targeted HRPs. Co-production, storytelling and flexible design emerged as promising components. Longer-term follow-up was rare.

**Conclusion:**

Evidence on inclusion training for HCPs demonstrates a range of approaches, such as co-production, reflective activities and flexible delivery, that may be relevant when considering training for HRPs. However, only one study targeted HRPs, highlighting an evidence gap. Future research is therefore needed to examine how such approaches can be adapted, implemented and evaluated within health research settings, including their theoretical grounding, contextual relevance and potential for longer-term impact.

**Supplementary Information:**

The online version contains supplementary material available at 10.1186/s12913-026-14263-1.

## Introduction

As the UK becomes increasingly diverse, it is vital for healthcare and health research professionals (HRPs) to develop strong cultural competency skills. This approach not only ensures better care for diverse populations, but also enhances the recruitment of representative research cohorts. Inclusive research practices generate evidence that informs policies and practices, ultimately reducing health inequalities [1, 2]. Beyond clinical care, HRPs help to shape study design, eligibility criteria, recruitment strategies, and participant communication, factors that directly influence whether research is inclusive and representative. Targeted training for HRPs therefore offers a meaningful opportunity to improve equity in both research participation and the conduct of studies. Despite this, such training is rarely described or evaluated in peer-reviewed literature.

Cultural competency is commonly defined as the ability to respect and understand another person’s attitudes, beliefs, values, behaviours and interpersonal styles associated with their culture [3]. Although the term remains prevalent in the titles and content of many training or education interventions, it is now widely agreed that no individual can be competent in all cultures. This shift has led to greater emphasis on concepts such as cultural humility [4], cultural sensitivity [5], and cultural safety [6]. These terms reflect a journey towards understanding one’s own beliefs and biases, while developing a collaborative, respectful and humble attitude towards others’ cultures. Thus, it is a lifelong journey of personal and professional development [7].

This review examined all forms of cultural competency, cultural competence, diversity and inclusion training, and broader related terms that encompass cross-cultural understanding and inclusive practices. This approach was necessary given the global variation in terminology related to cultural competency and inclusion. In addition to delivering culturally competent care, many healthcare professionals (HCPs) are also actively involved in health research. Health research underpins the evidence base for treatments and interventions used across clinical practice. However, participation by people from underserved communities, who face barriers to taking part in research, remains disproportionately low [8]. These barriers may include language difficulties, financial or logistical limitations, cultural exclusion, and belonging to groups that have historically been marginalised or overlooked in research design and recruitment. The reasons for limited participation are complex and multifactorial, but frequently include distrust of medical institutions, limited English proficiency, and concerns regarding the additional time and burden associated with participation [9]. While some organisations may offer internal inclusion training for HRPs, such programmes are seldom published or formally evaluated. This review therefore focuses on the available literature to identify effective components that can inform the development of a dedicated, evidence-informed training package to support ongoing training and development for HRPs.

### Aim

This systematic review aimed to map and synthesise published literature on cultural competency and other diversity and inclusion training for HCPs, and to identify elements that may be relevant for the development of future inclusion training for HRPs.

### Review questions

What types of cultural competency or other diversity and inclusion training programmes have been delivered to HCPs (and where available, HRPs) What were their objectives, methods, and outcomes? Which components could be utilised or adapted to help inform the development of a tailored inclusion training programme for HRPs?

## Methods

A protocol for this review was developed in advance and is publicly available via the University of Leicester, Leicester Research Archive 10.25392/leicester.data.28284974.v1 The review was not registered with PROSPERO because it does not focus on clinical effectiveness or health outcomes, which are required for registration in that database. The review was originally planned as a scoping review, but was finalised as a systematic review to enable the use of pre-specified eligibility criteria, dual independent screening, duplicate data extraction and structured quality appraisal using Joanna Briggs Institute JBI checklists, while retaining a structural narrative synthesis appropriate to the heterogeneity of training interventions, with study quality informing interpretation of findings. It was reported according to the PRISMA guidelines [10].

### Information sources and search strategy

This review examined all forms of diversity and inclusion training, including cultural competency and related approaches that promote cross-cultural understanding and inclusive practices. Broad search terms, such as cultural competency, cultural competence, inclusion training and related concepts like diversity, equity, and inclusion (DEI), anti-racism; cultural safety; cultural sensitivity and unconscious bias training were used to capture a wide range of educational interventions. This inclusive strategy accounted for global variation in terminology and ensured comprehensive coverage of international literature aimed at improving responsiveness to diversity in healthcare and research. Studies employing any pedagogical method, qualitative, quantitative, or mixed methods, were included if they met the inclusion criteria. Training delivered as part of undergraduate or pre-registration medical or nursing curricula was excluded, as the review focussed on continuing professional development for practising professionals.

**Table 1:** Inclusion/exclusion criteria

	Population	Intervention	Outcome	Language	Literature Type
Inclusion Criteria	Healthcare or health research professionals	Cultural competency or other named inclusion or diversity training programme	Includes evaluation or other measures of effectiveness		
Exclusion Criteria	Training is part of a medical or nursing curriculum	Training focused on a specific disease area	No evaluation or effectiveness measure	Not written in English	Systematic or Scoping Review

These criteria focused on interventions targeting individuals involved in healthcare delivery or health research. For the purposes of this review, healthcare professionals (HCPs) refer to clinicians, nurses, allied health professionals and healthcare support staff. The term health research professionals (HRPs) include clinical and non-clinical researchers, and any study-related support staff, working on any form of healthcare research across all disease areas. We acknowledge that in practice, some individuals may occupy overlapping roles as both healthcare professionals and health research professionals. However, for clarity, these terms are used to distinguish the primary focus of training interventions and participant populations reported in the literature.

This review was limited to published studies and does not include internal or organisational training programmes that may not be publicly available or formally evaluated. Training embedded within medical or nursing student curricula was also excluded, as the focus of this review was on continuing professional development for the existing HCPs rather than pre-service education. This ensured relevance to professionals currently engaged in healthcare delivery and research.

Bibliographic searches for this review were completed by December 2024. We selected SCOPUS and Web of Science for multidisciplinary coverage and citation tracking, Ovid Medline for biomedical indexing (overlapping with PubMed), and CINAHL for nursing and allied health. This combination provided complementary coverage and reduced duplication while capturing interventions across clinical, education and implementation journals. Search years were set to the default for each database to ensure that the search was as wide as possible (1970–2024). Published literature included in the screening process underwent forward and reverse citation searching to identify any missing papers. An example of the full search strategy used for Web of Science is provided in Supplementary File 1.

### Source of evidence selected, data extraction and synthesis approach

Outputs from the database searches were imported into the online screening tool Rayyan [11]. Titles and abstracts were screened independently in duplicate by EB and AC against the predefined eligibility criteria (Table 1.). Full texts were screened independently in duplicate by EB and KA. The final list of included studies was agreed upon by all authors. Discrepancies at either stage were resolved through discussion. Reasons for exclusion at each stage were documented using a PRISMA flow diagram [10]. Data extraction was conducted independently in duplicate by EB and AC using a pre-developed data extraction template. Extracted data included study design, training content, delivery format, theoretical underpinning, participant population, evaluation methods, and reported outcomes. Data were compared, and any discrepancies were resolved through discussion with LG, EM and ND.

To interpret and compare findings across studies, this review employed a thematic narrative synthesis approach. A predefined thematic framework, developed in line with the review objectives, structured the synthesis. The main deductive themes included design and delivery of training interventions, use of theoretical frameworks or cultural competency models, evaluation method, and learning outcomes [12]. To allow for flexibility and the identification of new or novel insights, inductive coding was also applied. This enabled the inclusion of emerging themes such as participatory storytelling, intersectionality, digital delivery models and equity considerations [13]. Studies were grouped across themes and compared systematically using a comparative matrix, highlighting both convergence and divergence in findings. This structured synthesis allowed exploration of which components, such as duration, delivery format, partner involvement, or evaluation tools, were associated with more favourable or sustained training outcomes. During synthesis we sensitively weighted interpretations towards studies with stronger JBI ratings, particularly where multiple studies addressed similar components with varying methodological quality.

### Quality appraisal

Quality appraisal was conducted using the Joanna Briggs Institute (JBI) Critical Appraisal Checklists, matched to the study design of each included publication. This included the use of Randomised Controlled Trials, Quasi-Experimental Studies, and Qualitative Research Checklists. Each study was independently appraised in duplicate by two reviewers (EB and KA) using the full set of checklist criteria. Each study was assigned ratings of “Yes”, “No”, or “Unclear” to each checklist item [14], and any discrepancies were resolved through discussion with all authors. The quality appraisal findings were used to inform the synthesis, highlighting potential risk of bias or methodological limitations in the evidence [15].

## Results

### Study selection

**Fig. 1:**
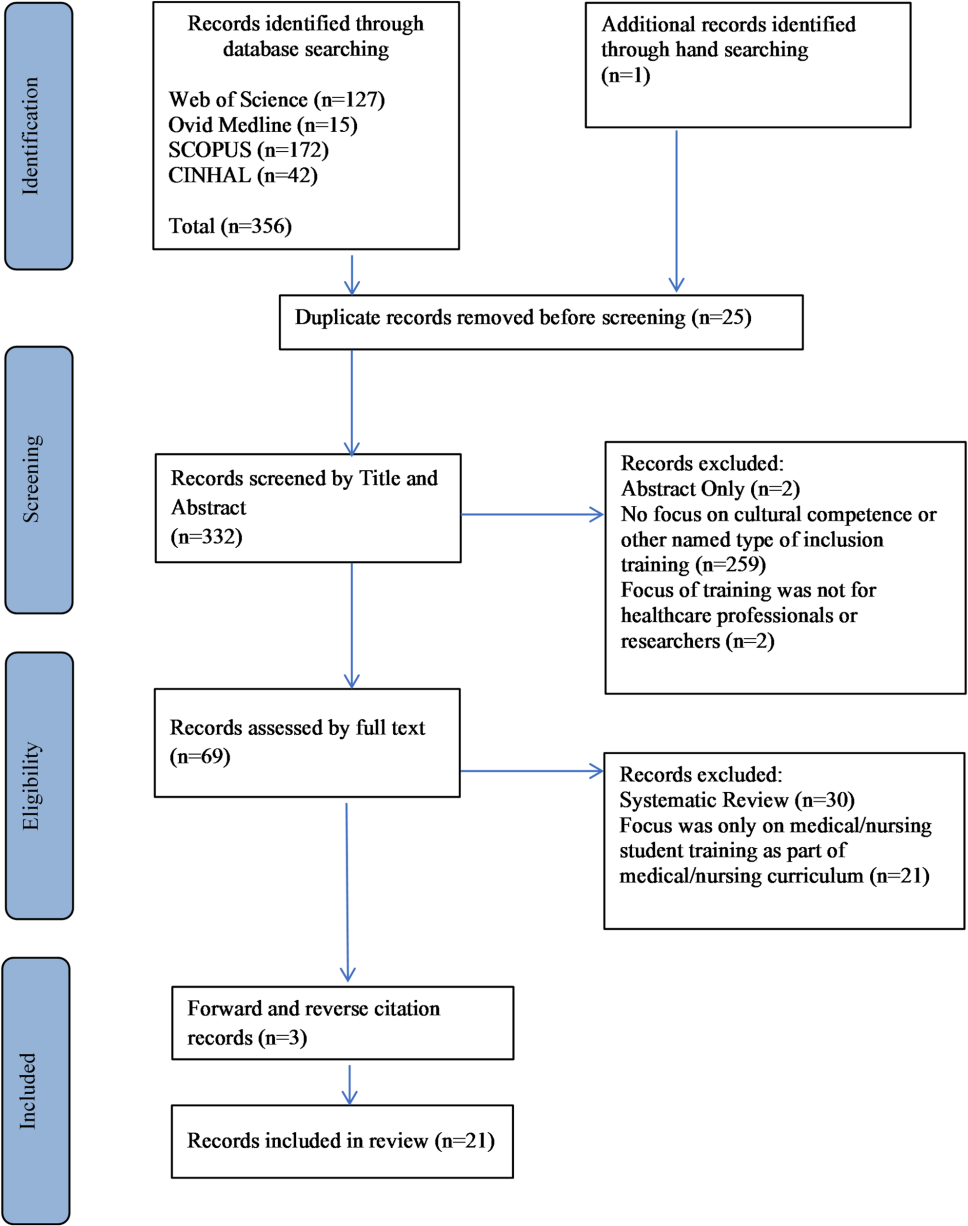
PRISMA flow diagram of study selection

The searches identified a total of 355 publications for screening. After removing duplicates, 330 were screened by title and abstract. The full texts of 67 articles were reviewed, with 51 excluded for being systematic reviews or for delivering training as part of a medical or nursing school curriculum. A final total of 21 publications was included in this systematic review with the inclusion and exclusion process described below (Fig. 1).

### Study characteristics

Of the 21 publications reviewed, 10 (47.6%) originated from European countries (including six from the UK). Nine studies (42.9%) were conducted in the USA, and one each (4.8%) from Canada and Australia. The papers ranged from 2003 to 2024; 33% (7/21) were published before 2010, 43% (9/21) between 2010 and 2020 and 24% (5/21) from 2021 to 2024. The included studies assessed training that had been implemented across a variety of healthcare settings and professional groups.

See Study Characteristics in Supplemental File 2.

Several studies involved multidisciplinary teams working in community or emergency care contexts. Participants commonly included nurses, physicians, mental health practitioners, pharmacists, and administrative or wider support staff. Only one study specifically targeted HRPs [16]. All other studies focused on training delivered to HCPs engaged primarily in clinical service delivery. While it is possible that additional inclusion training for HRPs is delivered within organisations, such activities are seldom reported in peer-reviewed literature, consequently the evidence base reflects a published literature gap rather than a definitive absence of practice.

Evaluation approaches varied considerably across studies but most sought to assess short-term changes in knowledge, attitudes, or self-reported behaviours. Seventeen studies reported improvements in at least one of these domains following training [5, 17–26]. In contrast, four studies either reported no notable changes or provided insufficient outcome data to draw firm conclusions [2, 27–29]. Evaluation methods predominantly relied on pre- and post-training assessments, using a combination of validated instruments and bespoke questionnaires. Validated tools included the Inventory for Assessing the Process of Cultural Competence among Healthcare Professionals Revised (IAPCC-R) [20], the Cultural Competence Assessment Tool (CCATool) [28, 30], and the Cross-Cultural Competence of Healthcare Professionals (CCCHP) [27]. Several studies complemented quantitative measures with qualitative feedback to support interpretation of findings [16, 18, 22, 24, 31, 32].

### Quality assessment

The overall methodological quality of the studies was acceptable. Of the three qualitative studies, two were rated high quality [22, 32], while one was judged to be of lower quality due to limited reporting [29]. Both randomised controlled trials were rated moderate quality, with limitations including participant drop-out and lack of blinding [5, 25]. The remaining 16 studies were assessed using the JBI checklist for quasi-experimental designs. Thirteen were rated as moderate quality, and three as high quality [20, 28, 33,]. In the findings, greater weight was therefore given to evidence from studies assessed a high quality.

### Design and delivery of training

Training delivery formats varied widely across the 21 studies. Eight interventions (38.1%) were delivered face-to-face, three (14.3%) were delivered entirely online and ten (47.6%) used hybrid or blended approaches. Prior to 2009, all training was delivered in-person. The first distance-learning format was reported in 2009 [29]. More recent studies increasingly adopted online or blended models [16, 17, 19, 24].

Training duration and structure varied widely, ranging from brief one-off sessions to programmes delivered over several months. For example, one study delivered content over a six-week period [18], another used a series of five storytelling events [22], and one provided a structured four-part programme for research staff [16]. In contrast, several studies provided minimal detail on training content or delivery methods [5, 26, 27]. Across studies that described their interventions clearly, common approaches included group discussions, case studies, role-play, reflective exercises, and interactive workshops aimed at improving cultural awareness, improving diversity knowledge and fostering inclusive communication.

### Target audience

Only one study specifically targeted HRPs [16]. All other studies targeted HCPs from a range of clinical settings, Participant cohorts ranged from five to 135. Two studies delivered training across multiple sites and professional groups, highlighting the importance of tailoring content to differing roles and organisational contexts [26, 33].

Four studies incorporated comparison or control groups [27, 33–35]. While some reported improvements in knowledge or awareness, findings were inconsistent and influenced by practical barriers such as workload pressures and limited institutional support.

HCPs were the predominant participant group across the evidence base. Few studies disaggregated outcomes by gender, and non-explicitly addressed gender inclusion within research participation. Although this was not a focus of the present review, the limited attention to gender-related considerations represents a gap for future work [36].

### Recognised model used

Five studies explicitly drew on established cultural competency models. Three studies applied the Papadopoulos, Tilki and Taylor Model (1998) [2, 28, 29, 37]. One used the Camphina-Bacote Model (1991) [31, 38], and another based their training on the Purnell Model [19, 39]. These models provided theoretical grounding and supported structured evaluation. Two further studies adapted existing models [27, 32], while others developed context-specific or pragmatic approaches without reference to a formal model [5, 16–18, 20–24, 26, 33, 40]. Overall, approximately half of the studies did not clearly report a theoretical underpinning.

### Partner engagement and collaborative input

Approaches to community and patient involvement varied considerably. Only one study fully employed co-production, involving LGBTQ+ community members as equal partners in designing, delivering, and evaluating training [22]. Other studies incorporated more limited forms of consultation, such as engaging community facilitators or expert panels [18, 28]. Evaluations suggested that interventions involving lived-experience perspectives were perceived positively by participants [22], although the overall evidence base remains limited.

### Evaluation & evaluation tools

Most studies assessed outcomes immediately after training. Nine studies (~ 43%) used at least one validated measurement tool, while the remainder relied on bespoke questionnaires or reflective exercises.

A summary of the evaluation tools used across studies is provided in Table 2.

**Table 2:** Evaluation tools

Study Authors	Tool Name	Purpose/Focus
Delgado et al. [20]	IAPCC-R	Measure changes in cultural competence among healthcare professionals
Papadopoulos et al. [2, 28]	Competence Assessment Tool (incl. child mental health version) (CCATool)	Assess cultural competence, including a version for child mental health settings
Owiti et al. [23]	Modified TACCT	Assess cultural competence training using a modified AAMC tool
Thom et al. [25]	Patient satisfaction and physician cultural competence surveys (PRPCC)	Gathers patient feedback on physician cultural competence
Bristol et al. [17]	Ally Identification Measure	Evaluates LGBTQ+ Ally identity
Long, et al. [22]	Gay-Affirmative Practice Scale	Assesses attitudes and practices towards LGBTQ+ individuals
Beck et al. [27]	CCHCP	Measures cross-cultural competence of healthcare professionals
Majumdar et al. [5]	Rokeach Dogmatism Scale	Assess openness to new cultural information

Seventeen studies reported positive short-term changes in cultural awareness, knowledge, and self-reported confidence. However, evidence of long-term behavioural change was limited. Only six studies included follow-up beyond the immediate post-training period, often with low response rates. Where longer-term follow-up was conducted, results were mixed, with some studies noting partial retention of knowledge [20, 31], and others finding no sustained improvement [25, 27].

Only four studies used control or comparison groups, and many evaluations relied on self- reported measures. Outcomes appeared stronger in programmes that were interactive, modular and tailored to specific participant groups. The evidence base largely reflects short-term educational outcomes among HCPs, with only one study directly evaluating training for HRPs [16]. Consequently, the applicability of these findings to HRPs must be interpreted cautiously.

## Discussion

This systematic review synthesised evidence from 21 studies evaluating cultural competency and inclusion training delivered primarily to HCPs. Although most studies reported short-term improvements in knowledge, attitudes and self-reported behaviours, these outcomes were largely immediate and based on self-reported measures, suggesting that the current evidence is more informative about how training is experienced by participants than how it influences sustained professional practice. These findings therefore provide indirect rather than direct evidence to inform training for HRPs. Only one included study specifically targeted this group, highlighting a substantial gap in the published literature. Consequently, conclusions regarding the health research workforce must be interpreted cautiously and understood as exploratory and agenda-setting rather than definitive. While the synthesis highlights gaps and variations across the literature, these observations arise from systematic identification, appraisal and comparative interpretation of studies, rather than from exploratory mapping alone.

The studies included employed a wide range of methodological approaches. Only a small number used quasi-experimental designs with comparison or control groups [27, 34], while most relied on pre-and post-intervention assessments without controls [16, 19, 24, 26, 40]. Although such designs are common in applied training contexts, they limit the ability to draw causal inferences regarding effectiveness, particularly in relation to longer-term or organisational-level outcomes. Additional methodological limitations included variable follow-up periods, attrition, and limited analysis of drop-out or non-response [17, 19, 24]. Taken together, these factors reduce the overall strength and generalisability of the evidence base.

Since formal quality appraisal was undertaken, it is important to interpret findings in light of study quality. Evidence from studies rated as high quality generally reported positive short-term outcomes. However, even these studies rarely assessed sustained behavioural or organisational change. Lower-quality studies were more likely to rely solely on self-report measures and lacked detailed evaluation frameworks. This suggests that, while training can influence immediate learning outcomes, the strength of current evidence for longer-term impact remains limited. Where similar outcomes were reported across studies of varying methodological quality, greater interpretive weight was given to findings from studies assessed as higher quality.

Limited attention to researcher reflexivity was evident across several qualitative and mixed-methods studies. In a number of cases, authors did not fully describe their positionality or consider how this might have influenced data collection and interpretation [16, 22, 23, 29]. While this does not invalidate the findings, greater transparency regarding reflexive processes would have strengthened the credibility and depth of the evaluations, particularly where training addressed issues of identity, power or structural inequality.

While immediate post-training feedback was generally positive, evidence of longer-term impact was limited. Only six studies assessed outcomes beyond the immediate post-intervention period and follow-up rates were often low. Some studies reported partial knowledge retention at three to 12 months [20, 22, 23], whereas others found no sustained improvement [25, 27]. These mixed findings indicate that short-term gains in awareness or attitudes do not necessarily translate into enduring changes in practice. This distinction is important when considering how training outcomes are conceptualised and evaluated within the literature. They also highlight the importance of organisational support and follow-up mechanisms if training is to have meaningful long-term impact [35].

Over time, training content and delivery methods have evolved from predominantly lecture-based approaches towards more interactive and participatory formats [22, 24, 27]. Recent interventions have incorporated hybrid formats, digital platforms and storytelling techniques and have increasingly address broader issues like intersectionality, implicit bias and structural health inequities. Thus, reflecting a broader shift from traditional cultural competency towards frameworks of cultural humility, cultural safety and anti-racism [4, 6, 41]. Despite these developments, many programmes lacked clear theoretical grounding or explicit links between learning objectives and evaluation methods. The absence of consistent theoretical frameworks makes it difficult to determine which components are most likely to drive change or make meaningful comparisons across interventions.

Although the evidence base relates predominantly to HCPs rather than HRPs, several potentially transferable principles emerge from the review. Interventions that were interactive, reflective and tailored to specific participant groups were more consistently associated with positive short-term outcomes. Approaches that incorporated lived-experience perspectives, community involvement or participatory learning were also viewed favourably by participants. While these elements have not yet been formally evaluated within a HRP cohort, they provide a set of practical design characteristics that may inform the development of future training for HRPs. However, in the absence of direct evaluation within research settings, these principles should be regarded as provisional.

Participant engagement and retention were recurrent challenges across studies. Low follow-up rates and competing clinical demands limited many evaluations, particularly for voluntary or online programmes. These findings suggest that future initiatives would benefit from careful integration within organisational structures, protected time for participation, and planned follow-up assessments. Such observations should therefore be viewed as hypotheses for future research rather than established evidence-based solutions.

The limited evidence base for HRPs presents particular challenges. Training needs for this group may differ from those of HCPs, given their work across varied areas, from clinical and translational studies to laboratory science, population health, and community-based research. However, with only one published programme focused on this population, there is insufficient evidence to determine which approaches are most appropriate. Future research should therefore prioritise the development and rigorous evaluation of interventions specifically tailored to HRPs.

An additional observation from this review was the limited attention to gender-related considerations within inclusion training. Few studies reported gender-disaggregated outcomes, and none explicitly examined how the recruitment or engagement of women or other wider under-represented groups in health research. This represents a gap in the current literature rather than a conclusion that can be drawn from the evidence reviewed. As such, it reflects limitations in reporting and study focus rather than evidence of absence. Further research would be needed to explore how issues of sex, gender and intersectionality might be more systematically incorporated into future training programmes.

This review offers a unique contribution by examining how evidence from training delivered to HCPs might inform future training initiatives for HRPs. However, given that the available evidence is largely short-term, methodologically heterogeneous and not directly focused on HRPs, the findings should be interpreted as highlighting potential directions rather than providing firm guidance. There remains a clear need for targeted, theory-informed and rigorously evaluated interventions designed specifically for HRPs.

This review has several limitations. It included only peer-reviewed studies published in English, potentially excluding relevant interventions in other languages or grey literature. Consequently, no studies were identified from low and middle-income countries or non-English speaking regions. This likely reflects the inclusion criteria rather than a lack of activity, thus limiting global generalisability. Future reviews would benefit from multilingual search strategies to better capture international practice.

The included studies varied widely in design, participants, content, delivery and evaluation methods, limiting quantitative synthesis and generalisability. Some lacked methodological detail, hindering comparison and critical appraisal. Measurement reliability also varied: although many used validated tools (e.g., IAPCC-R, CCATool, CCCHP), others relied on unvalidated instruments [21, 24]. Most studies focused on short-term outcomes, with few assessing long-term behavioural impact. Publication bias may also be present, as studies with null or negative findings are less likely to be published [42]. Despite these limitations, the overall quality was acceptable and the findings offer valuable insights into the design and delivery of cultural competency and inclusion training in healthcare. Additionally, because only one study focused directly on HRPs, the applicability of the findings to this group is limited.

## Conclusion

This review indicates that cultural competency and inclusion training delivered to HCPs can support short-term improvements in knowledge, attitudes and awareness. However, the evidence base is characterised by methodological heterogeneity, limited long-term follow-up and a reliance on self-reported outcomes. Importantly, only one published study specifically targeted HRPs, highlighting a gap in the peer-reviewed literature. The findings therefore offer indirect rather than definitive guidance for the development of bespoke training for HRPs. While there is insufficient evidence to determine which training components are most effective for HRPs or to demonstrate sustained impact on practice, the review identifies a set of potentially transferable principles from HCP training, such as interactive and reflective learning, participatory design and context-specific delivery, that may provide a useful starting point for developing bespoke training for HRPs.

Future research should prioritise the design and rigorous evaluation of inclusion training specifically tailored to HRPs. Such work would benefit from clear theoretical foundations, robust evaluation, and ideally longer-term follow-up to assess behavioural and organisational outcomes. Although several included studies addressed aspects of diversity, such as LGBTQ+ inclusion or culturally specific needs, most did not report gender‑disaggregated outcomes or apply explicit intersectional analytical frameworks. These areas therefore represent underdeveloped dimensions of the published evidence rather than findings emerging directly from the interventions. Future research could explore how gender and intersectionality are incorporated into the design and evaluation of training programmes, as this was only variably addressed across the reported literature. Overall, this review underscores a clear need for high-quality, context-specific research focused on inclusion training for HRPs. Developing and evaluating such interventions represents an important next step towards strengthening equitable and inclusive health research practice.

## Supplementary Information

Below is the link to the electronic supplementary material.

Supplementary Material 1: Table. Search Strategy (Web of Science)

Supplementary Material 2: Table. Study Characteristics

## Data Availability

All data supporting the findings of this study are included in the manuscript and supplementary materials.

## References

[1] Brown G, Marshall M, Bower P, Woodham A, Waheed W. Barriers to recruiting ethnic minorities to mental health research: a systematic review. 2014;23:36–48. 10.1002/mpr.1434. 10.1002/mpr.1434 PMC6878438 24474683

[2] Papadopoulos I, Tilki M, Lees S. Promoting cultural competence in healthcare through a research based intervention in the UK. 2004;1. https://www.primescholars.com/articles/promoting-cultural-competence-in-healthcare-through-a-research-based-intervention-in-the-uk.pdf.

[3] National Institute of Health National Library of Medicine. improving cultural competence 2014. https://www.ncbi.nlm.nih.gov/books/NBK248433/.

[4] Lekas H-M, Pahl K, Fuller Lewis C. Rethinking Cult Competence: Shifting Cult Humility. 2020;13:1178632920970580. 10.1177/1178632920970580. 10.1177/1178632920970580 PMC7756036 33424230

[5] Majumdar B, Browne G, Roberts J, Carpio B. Effects of cultural sensitivity training on health care provider attitudes and patient outcomes. 2004;36:161–6. 10.1111/j.1547-5069.2004.04029.x. 10.1111/j.1547-5069.2004.04029.x 15227764

[6] Cox JL, Simpson MD. Cultural Humility: A Proposed Model for a Continuing Professional. Dev Program. 2020;8:214. 10.3390/pharmacy8040214. 10.3390/pharmacy8040214 PMC7712005 33202754

[7] Stubbe DE. Practicing cultural competence and cultural humility in the care of diverse patients. 2020;18:49–51. 10.1176/appi.focus.20190041. 10.1176/appi.focus.20190041 PMC7011228 32047398

[8] National Health Service (NHS). England. Increasing diversity in research participation. 2023. https://www.england.nhs.uk/aac/publication/increasing-diversity-in-research-participation/.

[9] Heller C, Balls-Berry JE, Nery JD, Erwin PJ, Littleton D, Kim M, et al. Strategies addressing barriers to clinical trial enrolment of underrepresented populations: A. Syst Rev. 2014;39:169–82. 10.1016/j.cct.2014.08.004. 10.1016/j.cct.2014.08.004 PMC6936726 25131812

[10] Page MJ, McKenzie JE, Bossuyt PM, Boutron I, Hoffmann TC, Mulrow CD, et al. The PRISMA 2020 statement: an updated guideline for reporting systematic reviews 2021;372:n71–71. 10.1136/bmj.n71. 10.1136/bmj.n71 PMC8005924 33782057

[11] Ouzzani M, Hammady H, Fedorowicz Z, Elmagarmid A. Rayyan–a web and mobile app for systematic reviews. 2016;5. 10.1186/s13643-016-0384-4. 10.1186/s13643-016-0384-4 PMC5139140 27919275

[12] Carroll C, Booth A, Leaviss J, Rick J. Best fit. Framew synthesis: Refin method. 2013;13:37. 10.1186/1471-2288-13-37. 10.1186/1471-2288-13-37 PMC3618126 23497061

[13] Thomas J, Harden A. Methods for the thematic synthesis of qualitative research. Syst reviews. 2008;8:45–45. 10.1186/1471-2288-8-45. 10.1186/1471-2288-8-45 PMC2478656 18616818

[14] Joanna Briggs Institute checklist for. randomized controlled trials, checklist for quasi-experimental studies, checklist for qualitative research 2017. https://jbi.global/critical-appraisal-tools.

[15] Aromataris. JBI Manual for Evidence Synthesis. 2024. 10.46658/jbimes-24-01.

[16] Hill Weller L, Rubinsky AD, Shade SB, Liu F, Cheng I, Lopez G, et al. Lessons learned from implementing a diversity, equity, and inclusion curriculum for health research professionals at a large academic research institution. 2024;8. 10.1017/cts.2024.6. 10.1017/cts.2024.6 PMC10879992 38384906

[17] Bristol S, Kostelec T, Macdonald R. Improving Emergency Health Care Workers’ Knowledge, Competency, and Attitudes Toward Lesbian, Gay, Bisexual, and Transgender Patients Through. Interdisciplinary Cult Competency Train. 2018;44:632. 10.1016/j.jen.2018.03.013. 10.1016/j.jen.2018.03.013 29704979

[18] Chapman R, Martin C, Smith T. Evaluation of staff cultural awareness before and after attending cultural awareness training in an Australian. Emerg department. 2014;22:179. 10.1016/j.ienj.2013.11.001. 10.1016/j.ienj.2013.11.001 24412133

[19] Debiasi LB, Selleck CS. Cultural Competence Training for Primary Care Nurse Practitioners: An Intervention to Increase Culturally. Competent Care. 2017;24:39–45.

[20] Delgado DA, Ness S, Ferguson K, Engstrom PL, Gannon TM, Gillett C. Cult Competence Train Clin Staff. 2013;24:204. 10.1177/1043659612472059. 10.1177/1043659612472059 23389745

[21] Donisi V, Amaddeo F, Zakrzewska K, Farinella F, Davis R, Gios L, et al. Training healthcare professionals in LGBTI cultural competencies: exploratory findings from the Health4LGBTI pilot project. 2020;103:978–87. 10.1016/j.pec.2019.12.007. 10.1016/j.pec.2019.12.007 31866197

[22] Long A, Jennings J, Bademosi K, Chandran A, Sawyer S, Schumacher C, et al. Storytelling to improve healthcare worker understanding, beliefs, and practices related to LGBTQ + patients: A program evaluation. 2022;90:101979. 10.1016/j.evalprogplan.2021.101979. 10.1016/j.evalprogplan.2021.101979 34275639

[23] Owiti JA, Ajaz A, Ascoli M, De Jongh B, Palinski A, Bhui KS. Cultural consultation as a model for training multidisciplinary mental healthcare professionals in cultural competence skills. preliminary results. 2013;21:814. 10.1111/jpm.12124. 10.1111/jpm.12124 24279693

[24] Roberts KJ, Omaits E. Evaluation of a Virtual Health Equity Training for Mid-Career Primary. Healthc Providers. 2023;10:23821205231219616. 10.1177/23821205231219614. 10.1177/23821205231219614 PMC10729640 38116494

[25] Thom DH, Tirado MD, Woon TL, Mcbride MR. Development and evaluation of a cultural competency training curriculum. 2006;6. 10.1186/1472-6920-6-38. 10.1186/1472-6920-6-38 PMC1555583 16872504

[26] Webb E, Sergison M. Evaluation of cultural competence and antiracism training in child health services. 2003;88:4:291–4 10.1136/adc.88.4.291. 10.1136/adc.88.4.291 PMC1719527 12651748

[27] Beck P, Matusiewicz D, Schouler-Ocak M, Khan Z, Peppler L, Schenk L. Evaluation of cross-cultural competence among German health care professionals: A quasi-experimental study of training in two hospitals. 2024;10. 10.1016/j.heliyon.2024.e27331. 10.1016/j.heliyon.2024.e27331 PMC10950504 38509980

[28] Papadopoulos I, Tilki M, Ayling S. Cultural competence in action for CAMHS: Development of a cultural competence assessment tool and. Train programme. 2008;28:129–40. 10.5172/conu.673.28.1-2.129. 10.5172/conu.673.28.1-2.129 18844566

[29] Papadopoulos I, Kelly F. Enhancing the cultural competence of healthcare professionals through an online course. 2009;6:77–84 https://www.primescholars.com/articles/enhancing-the-cultural-competence-of-healthcare-professionals-through-an-online-course.pdf.

[30] Seeleman C, Suurmond J, Stronks K. Cultural competence: A conceptual framework for teaching and learning. Med Educ. 2009;43(3):229–237. 10.1111/j.1365-2923.2008.03269.x. 10.1111/j.1365-2923.2008.03269.x 19250349

[31] Brathwaite AC, Majumdar B. Evaluation of a cultural competence educational programme. 2006;53:470–9. 10.1111/j.1365-2648.2006.03742.x. 10.1111/j.1365-2648.2006.03742.x 16448490

[32] Kaihlanen A-M, Hietapakka L, Heponiemi T. Increasing cultural awareness: qualitative study of nurses’ perceptions about cultural competence training. 2019;18:38–9. 10.1186/s12912-019-0363-x. 10.1186/s12912-019-0363-x PMC6704569 31440116

[33] Celik H, Abma TA, Klinge I, Widdershoven GAM. Process evaluation of a diversity training program: The value of a mixed method strategy. 2011;35:54. 10.1016/j.evalprogplan.2011.07.001. 10.1016/j.evalprogplan.2011.07.001 22054525

[34] Prescott-Clements L, Schuwirth L, Van Der Vleuten C, Hurst Y, Whelan G, Gibb E, et al. Cult Competence Health Care Professionals. 2012;36:191. 10.1177/0163278712454137. 10.1177/0163278712454137 22843423

[35] Bastami F, Zamani-Alavijeh F, zareban I, Araban M. Explaining the experiences of health care providers regarding organizational factors affecting health education: a qualitative study. 2022;22:1–743. 10.1186/s12909-022-03807-8. 10.1186/s12909-022-03807-8 PMC9609166 36303173

[36] Abasi K. Under-representation of women in research: a status quo that is a scandal. British Medical Journal. Editor’s Choice. 2023 https://www.bmj.com/content/382/bmj.p2091.

[37] Papadopoulos I, Taylor G, Ali S, Aagard M, Popescu S, Ahmad W, et al. Developing tools to promote culturally competent compassion, courage and intercultural communication in healthcare. J Compassionate Health Care. 2016;3:2. 10.1186/s40639-016-0019-6.

[38] Campinha-Bacote J. Community mental health services for the underserved: A culturally specific model. 1991;5:229–35. 10.1016/0883-9417(91)90051-6. 10.1016/0883-9417(91)90051-6 1953048

[39] Purnell L. A Description of the Purnell Model for. Cult Competence. 2000;11:40–6. 10.1177/104365960001100107. 10.1177/104365960001100107 11982073

[40] Tillman F, Liu I, Lovince J, Mays E, Musick K, Sato J, et al. Healthcare Equity and Leadership: Implementation of Diversity, Equity, and Inclusion. Train Pharm Residents. 2024;37:422–8. 10.1177/08971900221142684. 10.1177/08971900221142684 36446745

[41] Tervalon M, Murray-Garcia J. Cultural Humility Versus Cultural Competence: A Critical Distinction in Defining Physician Training. Outcomes Multicultural Educ. 1998;9:117–25. 10.1353/hpu.2010.0233. 10.1353/hpu.2010.0233 10073197

[42] Pannucci CJ, Wilkins EG. Identifying and Avoiding Bias. Res. 2010;126:619–25. 10.1097/PRS.0b013e3181de24bc. 10.1097/PRS.0b013e3181de24bc PMC2917255 20679844

