# Supplementary material for: Cultural competency and inclusion training for healthcare professionals: a systematic review to inform training for health research professionals
Source: BMC Health Serv Res. 2026 Mar 3;26:481. doi: 10.1186/s12913-026-14263-1 (PMC13064406; doi:10.1186/s12913-026-14263-1)
Supplement: Supplementary file 1 — Supplementary Material 1: Table. Search Strategy (Web of Science) [file 12913_2026_14263_MOESM1_ESM.docx]

| **Database**  **Web of Science** | **Search Years**  **(1970-2024)** |
| --- | --- |
| 1: (((((((TS=("cultural competenc* training")) OR TS=("cultural sensitivity training")) OR  TS=("cultural humility training")) OR TS=("health equality training")) OR TS=("health equity  training")) OR TS=("diversity and inclusion training")) OR TS=("intercultural training")) OR  TS=("anti-racist training")  2: ((((((((TS=("cultural competenc* education")) OR TS=("cultural sensitivity education")) OR  TS=("cultural humility education")) OR TS=("health equality education")) OR TS=("health equity  education")) OR TS=("diversity and inclusion education")) OR TS=("intercultural education")) OR  TS=("anti-racist education")) OR TS=("antiracist education")  3: ((((((((TS=("cultural competenc* course")) OR TS=("cultural sensitivity course")) OR  TS=("cultural humility course")) OR TS=("health equality course")) OR TS=("health equity  course")) OR TS=("diversity and inclusion course")) OR TS=("intercultural course")) OR  TS=("anti-racist course")) OR TS=("antiracist course")  4: ((((((((TS=("diversity training")) OR TS=("diversity education")) OR TS=("diversity course"))  OR TS=("inclusion training")) OR TS=("inclusion education")) OR TS=("inclusion course")) OR  TS=("diversity program*")) OR TS=("diversity workshop*")) OR TS=("inclusion  workshop*")  5: (((((((TS=("cultural competenc* program*")) OR TS=("cultural sensitivity program*")) OR  TS=("cultural humility program*")) OR TS=("health equality program*")) OR TS=("health equity  program*")) OR TS=("intercultural program*")) OR TS=("anti-racist program*")) OR  TS=("antiracist program*")  6: (((((((TS=("cultural competenc* workshop")) OR TS=("cultural sensitivity workshop")) OR  TS=("cultural humility workshop")) OR TS=("health equality workshop")) OR TS=("health equity  workshop")) OR TS=("intercultural workshop")) OR TS=("anti-racist workshop")) OR  TS=("antiracist workshop")  7: #6 OR #5 OR #4 OR #3 OR #2 OR #1  8: (((TS=("medical research*")) OR TS=("clinical research*")) OR TS=("health research*")) OR  TS=("biomedical research*")  9: #7 AND #8  10: ((((((((((((TS=(effect*)) OR TS=(evaluat*)) OR TS=(impact*)) OR TS=(outcome*)) OR  TS=(measu*)) OR TS=(satisfaction*)) OR TS=(improved)) AND TS=(understanding)) OR  TS=(knowledg*)) OR TS=(awareness)) OR TS=("self-efficacy")) OR TS=(confidence)) OR  TS=(behavior*)  11: #9 AND #10  12: ((((((((((((TS=(healthcare)) OR TS=(health)) AND TS=(care)) OR TS=(medical)) OR  TS=(clinical)) OR TS=(researcher)) OR TS=(clinician)) OR TS=(provider)) OR TS=(doctor)) OR  TS=(physician)) OR TS=(nurse)) OR TS=("allied health")) OR TS=(professional)  13: #7 AND #12  14: #13 AND #10  Article or Review Article or Early Access or Book Chapters  (Document Types) and English (Languages) | |
