# Supplementary material for: Cultural competency and inclusion training for healthcare professionals: a systematic review to inform training for health research professionals
Source: BMC Health Serv Res. 2026 Mar 3;26:481. doi: 10.1186/s12913-026-14263-1 (PMC13064406; doi:10.1186/s12913-026-14263-1)
Supplement: Supplementary file 2 — Supplementary Material 2: Table. Study Characteristics [file 12913_2026_14263_MOESM2_ESM.docx]

| **Title** | **Author/s and publication year** | **Country** | **Participants** | **Framework/Model** | **Methods/Study Approach** | **Evaluation** | **Key Findings** |
| --- | --- | --- | --- | --- | --- | --- | --- |
| Evaluation of cultural competence and antiracism training in child health services | Webb & Sergison  (2003) | UK | Healthcare professionals from two sites.  Cardiff: (n=unspecified) Huddersfield (n=92) | None specified | Small group training using ERA pack. Duration and content unspecified. | Pre/post satisfaction questionnaires. With long-term follow-up at Cardiff: | Training increased cultural awareness and confidence. It was well received but not consistently prioritised. |
| Effects of cultural sensitivity training on health care provider’s attitudes and patient outcomes | Majumdar et al.,  (2004) | USA | Nurses and home care workers (n=114)  Patients (n=133) | None specified | RCT with 36-hour training for staff. Quantitative methods. | Multiple follow-up questionnaires using self-assessment and dogmatism scale. | Improved multicultural understanding among staff.  No significant patient outcome differences. |
| Promoting cultural competence in healthcare through a research based intervention in the UK | Papadopoulos  (2004) | UK | Mental health staff (unspecified) (n=35) | Papadopoulos, Tilki and Taylor Model (1998) | Eight work palace sessions over four months based on CCATool assessment | CCATool with VAS. Expert-validated. | Limited post-assessment completion. Suggested delayed evaluation and participatory programme design. |
| Evaluation of a cultural competence educational programme | Braithwaite  (2006) | Canada | Public Health Nurses (n=76) | Camphina-Bacote Model of Cultural Competence (2002) | Five weekly two-hour sessions plus booster, delivered via lectures, discussions and role play. | Repeat measures using 25-item cultural knowledge Likert scale. | Training improved cultural knowledge, supported by positive participant feedback. |
| Development and evaluation of a cultural competency training curriculum | Thom et al.,  (2006) | USA | Primary care doctors (n=53)  Primary care patients (n=429) | Co-author, Dr Miguel Tirado’s adapted model of cultural competence | Three-module training via half-day sessions. Mixed delivery methods. | PRPCC and seven-item patient satisfaction scale. | No improvement in cultural competence or patient experience. Limited institutional support noted. |
| Cultural competence in action for CAMHS; Development of a cultural competence assessment tool and training programme | Papadopoulos, Tilki & Ayling  (2008) | UK | CAMHS staff (clinical, nursing, AHP, admin)  (n=47) | Papadopoulos, Tilki and Taylor (1998) | Two-day Delphi model training using peer learning and reflection. | Pre/post CAMHS CCATool questionnaire. | Tool piloted and widely adopted. External evaluation ongoing. |
| Enhancing the cultural competence of healthcare professionals through an online course | Papadopoulos & Kelly  (2009) | UK | Midwives (exclusively female)  (n=5) | Papadopoulos, Tilki and Taylor (1998) | 150-hour online course over eight weeks with varied digital learning activities. | Weekly online questionnaires and final course evaluation. | Positive feedback on peer support and teaching. Small all-female sample noted as limitation. |
| Process evaluation of a diversity training program: The value of a mixed method strategy | Celik, Abama, Klinge & Widdershoven  (2011) | Netherlands | Healthcare staff across three settings (n=31) 60% female | None specified | Four-module programme across three healthcare settings. Delivered via group and individual activities. | Pre/post surveys on diversity awareness and satisfaction. Custom tool used. | Training more effective for mental health and hospital staff. Nursing home staff found it too academic. |
| The Cultural Competence of Health Care Professionals: Conceptual Analysis Using the Results From a National Pilot Study of Training and Assessment | Prescott-Clements et al.,  (2012) | UK | Vocational Dental Practitioners (n=76)  Control group (n=15) | Cultural competence framework for postgraduate vocational dental practitioners (framework validated in consultation with stakeholder groups) | Eight workshops using discussion and role play. Delivered by educators and stakeholders. | Workshop performance scored. Post-workshop evaluations and three-month follow-up. | Positive impact on practitioner behaviour. Caution advised on stakeholder-led session to avoid stereotyping. |
| Cultural Competence Training for Clinical Staff: Measuring the Effect of a One-Hour Class on Cultural Competence | Delgado et al.,  (2013) | USA | Nursing department staff (n=111) | None specified |  | IAPCC-R administered pre-training and at three and six-months. | Training improved cultural awareness. Slight decline at 6-months suggested a need for refresher training. |
| Evaluation of staff cultural awareness before and after attending cultural awareness training in an Australian emergency department | Chapman et al., (2014) | Australia | ED staff (n=72 invited) | None specified | Three two-hour workshops over six-weeks. Delivered via face-to-face, interactive and reflective methods. | Validated Aboriginal awareness survey. Pre/post training. | Training increased Aboriginal cultural awareness. Recommended broader studies. |
| Cultural consultation as a model for training multidisciplinary mental healthcare professionals in cultural competence skills: preliminary results | Owiti et al.,  (2013) | UK | Mental health professionals (n=94) | Cultural Consultation Service (CCS) Model Adapted from McGill Model of CCS-Canada | 15 Sessions plus in vivo practice. CCS model with ethnographic and narrative methods. | TACCT tool at baseline and seven-month follow-up. | Significant improvement in self-reported competence. Low follow-up response rated noted. |
| Cultural competence training for primary care nurse practitioners: An intervention to increase culturally competent care | Debiasi & Selleck  (2017) | USA | Nurse Practitioners (n=13) | Purnell Model for Cultural Competence | Tailored one-hour email delivered module with case studies and reflective questions. | CCA and modified client sensitivity survey pre/post training. | Improved awareness of stereotyping. Client feedback possibly biased. |
| Improving emergency health care workers’ knowledge, competency, and attitudes toward lesbian, gay, bisexual, and transgender patients through interdisciplinary cultural competency training | Bristol, Kostelec & MacDonald (2018) | USA | ED staff (n=135) | None specified | Flipped classroom with e-learning and two-hour facilitator-led sessions. | AIM survey pre/post tested across three subscales. | Training improved attitudes and knowledge. Gender imbalance noted. |
| Increasing cultural awareness: qualitative study of nurses' perceptions about cultural competence training | Kaihlanen, Hietapakka & Hepooniemi  (2019) | Finland | Registered nurses (n=14)  Practical nurses (n=6) | Cultural Awareness component of Camphina-Bacote Model | Four weekly, four-hour sessions using constructivist theory and storytelling. | Semi-structured interviews transcribed and thematically analysed. | Positive feedback across categories. Suggestion included web-based options and religious content. |
| Training healthcare professionals in LGBTI cultural competencies: Exploratory findings from the Health4LGBTI pilot project | Donisi et al.,  (2020) | EU | Healthcare professionals/support staff (n=110) | None specified | One-day or split-day training with four modules using interactive methods. | Pre/post Likert scale questionnaires. | Training improved LGBTI knowledge and attitudes. Adaptable for different settings. |
| Storytelling to improve healthcare worker understanding, beliefs, and practices related to LGBTQ + patients: a program evaluation | Long et al.,  (2022) | USA | Healthcare Workers and community members (n=56) | None specified | Five storytelling events with selected speakers sharing themed personal stories. | Post-event surveys and open-ended reflections thematically analysed. | Storytelling enhanced understanding and empathy. Participation bias noted. |
| Evaluation of a Virtual Health Equity Training for Mid-Career Primary Healthcare Providers | Roberts & Omaits (2023) | USA | Mid-career primary care providers (n=21) | Health Equity and Implicit Bias training developed by third party | Two-day virtual training using adult learning theory and behaviour change model. | Pre/post surveys assessing knowledge, attitude and reflection. | Significant gains in knowledge and attitudes. Suggestion for broader access and additional topics. |
| Evaluation of cross-cultural competence among German health care professionals: A quasi-experimental study of training in two hospitals | Beck et al.,  (2024) | Germany | Healthcare professionals  Intervention (n=25) Control (n=91) | Cross-Cultural Competence for Healthcare Professionals build on Sue and Sue Model (1982) | Cross-cultural training covering motivation, attitudes, skills and empathy. Methods of delivery and timescales unspecified. | CCCHP self-reported tool across three time points. | No overall improvement noted. Drop in social desirability scores may indicate unconscious bias. |
| Healthcare Equity and Leadership: Implementation of Diversity, Equity, and Inclusion Training for Pharmacy Residents | Tillman et al., (2024) | USA | Pharmacy residents (n=41) | None specified | Nine monthly DEI seminars with internal and external speakers. Delivered via mixed formats. | Anonymous surveys after each seminar and final series evaluation. | High perceived benefit. Mixed rating across topics, with some rated very high. |
| Lessons learned from implementing a diversity, equity and inclusion curriculum for health research professional at a large academic research institution | Hill Weller et al., (2024) | USA | Epidemiology and Biostatistics Staff (n=250)  51% faculty  41% staff  8% trainees | None specified | Four DEI workshops using didactic and experiential methods. Developed via needs assessment. | Pre-post workshop surveys. Feedback used to adapt sessions. | Increased DEI knowledge, especially among minority groups. Feedback supported interactive formats. |
